# Minnesota multiphasic personality inventory as related factor for post traumatic stress disorder symptoms according to job stress level in experienced firefighters: 5–year study

**DOI:** 10.1186/s40557-015-0067-y

**Published:** 2015-06-05

**Authors:** In-Sung Chung, Mi-Young Lee, Sung-Won Jung, Chang-Wook Nam

**Affiliations:** Department of Occupational and Environmental Medicine, Dongsan Medical Center, Keimyung University School of Medicine, Daegu, Republic of Korea; Department of Psychiatry, Dongsan Medical Center, Keimyung University School of Medicine, Daegu, Republic of Korea; Department of Internal Medicine, Dongsan Medical Center, Keimyung University School of Medicine, Daegu, Republic of Korea

**Keywords:** Firefighter, MMPI, PTSD, Job stress

## Abstract

**Objectives:**

As first responders to an increasing number of natural and manmade disasters, active-duty firefighters are at increased risk for physical and psychiatric impairment as reflected by high rates of posttraumatic stress disorder (PTSD). Because little is known about related factor with PTSD according to job stress level among firefighters, we assessed utility of the Minnesota Multiphasic Personality Inventory (MMPI) using 5-year medical surveillance.

**Methods:**

Data were analyzed from 185 male firefighters without psychiatric disease history and who at assessments in 2006 and 2011 completed all questionnaires on personal behaviors (including exercise, drinking and smoking habits) and job history (including job duration and department). MMPI, Events Scale-Revised-Korean version (IES-R-K) and Korean Occupational Stress Scale-Short Form (KOSS-SF) were used to screen for personality trait, PTSD symptom presence and job stress level, respectively. IES-R-K subgroups were compared using two-sample t- and χ2 tests, and factors influencing IES-R-K according to KOSS-SF were determined using uni- and multivariate logistic regression.

**Results:**

Mean age and job duration were higher in PTSD-positive than negative groups. In multivariate analysis, increased PTSD risk was associated with: job duration (Odds ratio (OR) = 1.064, 95 % CI 1.012–1.118) for firefighters overall; masculinity-femininity (OR = 5.304, 95 % CI 1.191–23.624) and job duration (OR = 1.126, 95 % CI 1.003–1.265) for lower job stress level; and social introversion (OR = 3.727, 95 % CI 1.096–12.673) for higher job stress level.

**Conclusions:**

MMPI relates with PTSD according to job stress level among experienced firefighters. Masculinity-femininity and social introversion were the strongest related factor for PTSD symptom development in low and high job stress levels, respectively.

## Introduction

Professional firefighters are exposed to toxic substances and relatively high levels of emotional shock and traumatic stress in their line of duty as first responders to the increasing number of natural and manmade disasters [1–4]. Although estimated point-prevalence rates of PTSD symptoms can be as high as 38.57 % for active-duty professional firefighters [5], there is no established management program in Korea to address long-term adverse health effects of exposure to harmful materials or psychiatric trauma.

Several studies have focused on factors predisposing to PTSD development including pre-trauma vulnerability as personality disposition, exposure to traumatic event and the post-disaster-derived measure of neuroticism [6–8]. Negative appraisals involving self, the world, and blame are characteristic of PTSD development and maintenance [9, 10]. In addition, lower overall social support and reported lower levels of perceived support from family and employers because of the nature of the job, in particular shift work, were significant factors particularly among experienced firefighters [11]. KOSS-SF was developed over a period of 2 years (2002–2004) and is considered unique and specific to screen for occupational stressors among Korean employees particularly those triggered by changes in local social and economic sectors and in the general cultural milieu [12]. Job stress estimated by KOSS-SF has been associated with clinical psychogenic factors in cross sectional studies of Korean firefighters [13–15].

The purpose of this study was to evaluate utility of MMPI as related factor with PTSD according to job stress level using medical surveillance information collected from experienced firefighters over a 5-year period.

## Materials and methods

### Study population

**Fig. 1:**
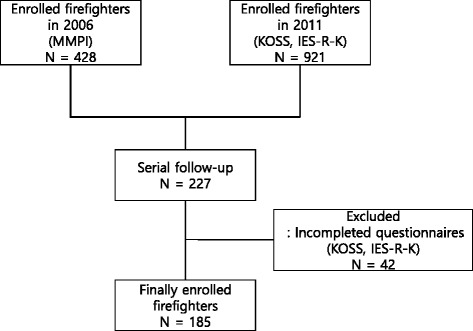
Flow chart for enrolled firefighters desposition

We collected data during annual health examinations for firefighters in 2006 and 2011. A total of 428 firefighters who answered MMPI in 2006 and 921 firefighters who assessed KOSS-SF and IES-R-K in 2011 were included in this study. Finally we analyzed data from 185 firefighters who had answered all administered questionnaires and provided written consent at the two assessment timepoints (Fig. 1). Questionnaires included personal behaviors (such as alcohol drinking, smoking, exercise) and job history (such as job duration and department). The study protocol was approved by the Institutional Review Board of Keimyung University Dongsan Medical Center (IRB File No. DSMC 2014-05-036-001).

### MMPI

MMPI included 556 items [16]. Using a computerized program, we produced converted score based on 3 validity and 10 clinical scales, and these K-corrected T scores were used for analysis. Cronbach’s α indicator of internal consistency was 0.786.

### KOSS-SF

Twenty-four questions on a four point Likert scale from zero (“not at all”) to four (“very much”) were included in KOSS-SF which was validated using factor analysis and standardized validation process by the National Study for Development and Standardization of Occupational Stress and included the following seven subscales: job demand (4 items, Cronbach’s α: 0.73), insufficient job control (4 items, Cronbach’s α: 0.59), inadequate social support (3 items, Cronbach’s α: 0.71), job insecurity (2 items, Cronbach’s α: 0.69), organizational system (4 items, Cronbach’s α: 0.78), lack of reward (3 items, Cronbach’s α: 0.73) and occupational climate (4 items, Cronbach’s α: 0.75) [12]. We divided each subscale into two groups, namely high and low, based on median subscale value. Total score calculated by sum of subscales was divided by 8.

### IES-R-K

IES-R-K, a validated measure of self-reported posttraumatic stress symptoms experienced in the past 7 days in relation to a specific stressor, includes 22 items in Korean language that measure symptoms of intrusion (dreams about the event), avoidance and numbing (effort to avoid reminders of the event), and hyperarousal (feeling watchful and on guard) with respect to particular life-threatening events [17]. Responders rate each item from zero (“not at all”) to four (“very much”), with total score ranging from 0 to 88. Screening positive for PTSD indicating the potential need for care and clinical assessment was indicated by a raw score of ≥18. Cronbach α was 0.98.

#### Statistical analysis

Data were analyzed using SPSS version 20. A two-sample t-test and χ2 test were used to examine differences between the two IES-R-K subgroups. Using uni- and multivariate logistic regression analysis with job duration as adjusting factor, we determined factors affecting IES-R-K scores stratified into KOSS-SF subgroups.

## Results

Data from 185 firefighters were finally included in this analysis. All study participants were male. General characteristics of workers in 2006 according to prevalence of PTSD subgroups are shown in Table 1. Mean age and job duration in positive subgroup (IES-R-K scores ≥18) were significantly higher than in negative subgroup. Other parameters were not significantly different between subgroups.

**Table 1:** General characteristics according to IES-R-K in 2006 (N = 185)

	IES –R-K		
	<18	≥18	*p*
Age^a^	38.32 ± 6.16	40.31 ± 6.21	0.039
Task (N/%)			0.446
Firefighting	64 (66.0)	33 (34.0)	
Emergency medical service	14 (53.8)	12 (46.2)	
Rescue	5 (45.5)	6 (54.5)	
Driver	13 (65.0)	7 (35.0)	
Desk work	24 (77.4)	7 (22.6)	
Job duration^a^			0.017
	11.68 ± 6.80	14.25 ± 7.05	
Smoking (%)			0.748
Smoker	16 (72.7)	6 (27.3)	
Ex-smoker	27 (64.3)	15 (35.7)	
Non-smoker	77 (63.6)	44 (36.4)	
Exercise (%)			0.516
No	19 (73.1)	7 (26.9)	
Yes	101 (63.5)	58 (36.5)	
Alcohol use (≥1 / week)			0.126
No	96 (66.7)	48 (33.3)	
Yes	24 (60.0)	17 (41.5)	

^a^Two sample t-test

Table 2 shows prevalence of PTSD according to job stress. Higher subgroups in job demand (p < 0.001), job insecurity (p < 0.001), organizational injustice (p = 0.013), occupational climate (p < 0.001) and sum (p < 0.001) had higher frequency of PTSD symptoms than lower subgroups.

**Table 2:** Prevalence of PTSD according to Job Stress in 2011

		IES-R-K		*p*
		<18	≥18	
KOSS				
Job demand	Low	55 (88.7)	7 (11.3)	0.000
	High	65 (52.8)	58 (47.2)	
Insufficient job control	Low	46 (66.7)	23 (33.3)	0.751
	High	74 (63.8)	42 (36.2)	
Interpersonal conflict	Low	7 (87.5)	1 (12.5)	0.264
	High	113 (63.8)	64 (36.2)	
Job insecurity	Low	42 (85.7)	7 (14.3)	0.000
	High	78 (57.4)	58 (42.6)	
Organizational injustice	Low	56 (75.6)	18 (24.3)	0.013
	High	64 (57.7)	47 (42.3)	
Lack of reward	Low	4 (100.0)	0 (0.0)	0.299
	High	116 (64.1)	65 (35.9)	
Occupational climate	Low	37 (88.1)	5 (11.9)	0.000
	High	83 (58.0)	60 (42.0)	
Sum	Low	82 (84.5)	15 (15.5)	0.000
	High	38 (43.2)	50 (56.8)	

Univariate and Multivariate analysis relating factors with PTSD symptom according to job stress level by job duration and personality traits are presented in Table 3. Overall, masculinity-femininity (OR = 2.234, 95 % CI: 1.194–4.180), social introversion (OR = 1.888, 95 % CI: 1.018–3.504) and job duration (OR = 1.055, 95 % CI:1.009–1.103) were significantly associated with PTSD symptoms. The higher subgroups in job demand (p < 0.001), job insecurity (p < 0.001), organizational injustice (p = 0.013), occupational climate (p < 0.001) and sum (p < 0.001) had higher frequency of PTSD symptoms. In multivariate analysis, increased risk for PTSD was associated with job duration (OR = 1.064, 95 % CI: 1.012–1.118) for firefighters overall, masculinity-femininity (OR = 5.304, 95 % CI: 1.191–23.624) and job duration (OR = 1.126, 95 % CI: 1.003–1.265) for lower job stress level, and social introversion (OR = 3.727, 95 % CI: 1.096–12.673) for higher job stress level.

**Table 3:** Univariate and Multivariate analysis relating factors with PTSD symptom according to job stress level

	Univariate						Multivariate					
	Total		KOSS_Low		KOSS_High		Total		KOSS_Low		KOSS_High	
	OR	95 % CI	OR	95 % CI	OR	95 % CI	OR	95 % CI	OR	95 % CI	OR	95 % CI
Personality traits (MMPI)												
Hypo-chondriasis	1.451	0.787–2.675	2.494	0.734–8.408	1.414	0.606–3.298	0.951	0.398–2.276	7.422	0.931–59.165	0.904	0.250–3.272
Depression	1.652	0.887–3.077	0.635	0.207–1.946	2.082	0.856–5.063	1.926	0.804–4.610	0.421	0.073–2.446	3.952	0.987–15.974
Hysteria	1.455	0.791–2.677	2.100	0.660–6.683	1.273	0.546–2.967	1.680	0.751–3.805	1.098	0.189–6.384	2.250	0.630–8.035
Psychopathic deviant	0.793	0.433–1.453	0.635	0.207–1.946	0.632	0.267–1.496	0.747	0.370–1.511	0.331	0.070–1.565	0.623	0.208–1.866
Masculinity-femininity	2.234	1.194–4.180	5.111	1.341–19.485	1.468	0.624–3.454	1.820	0.924–3.584	5.304	1.191–23.624	1.667	0.569–4.883
Paranoia	1.546	0.826–2.896	0.987	0.327–2.975	1.522	0.626–3.699	1.755	0.836–3.685	0.654	0.145–2.938	1.648	0.455–5.976
Psychasthenia	1.021	0.557–1.870	0.551	0.173–1.755	0.780	0.324–1.875	0.617	0.275–1.381	0.249	0.046–1.337	0.190	0.042–0.855
Schizophrenia	1.061	0.580–1.941	0.667	0.218–2.043	1.145	0.491–2.672	0.634	0.263–1.532	1.266	0.239–6.690	0.794	0.184–3.429
Hypomania	0.997	0.545–1.824	0.719	0.239–2.169	1.490	0.637–3.458	1.270	0.643–2.505	0.573	0.129–2.552	2.700	0.951–7.667
Social introversion	1.888	1.018–3.504	0.579	0.182–1.843	2.571	1.060–6.238	1.737	0.813–3.713	0.453	0.091–2.261	3.727	1.096–12.673
Job duration	1.055	1.009–1.103	1.085	0.999–1.178	1.049	0.983–1.118	1.064	1.012–1.118	1.126	1.003–1.265	1.042	0.961–1.130

## Discussion

This 5 years study showed MMPI, especially masculinity-femininity scale and social introversion, was significantly related with PTSD symptoms during chronic exposure to physical and mental health hazards in firefighters. In particular, this study assessed MMPI utility in prediction of PTSD stratified by job-related stress level among experienced firefighters. Preformed personality without intervention program for repeated exposure to trauma had been known to predispose to PTSD. In this study, masculinity-femininity and social introversion as well as job duration were important factors of PTSD development according to job stress level. In particular, social introversion was the most important factor of PTSD development in high job stress conditions. It is suggested that a continually strong social support system throughout a firefighter’s career is important to prevent social isolation in those with high stress level at work, particularly because even though firefighters have been examined annually since 2004 in South Korea, mental health evaluation was included in annual examination only after 2013 and thus far there has been no management program for firefighter’s mental issues.

Firefighters are regularly engaged in intensely traumatic events, including exposure to injuries or death and unpredictable dangerous situations. There is convergent evidence that less than one fourth of individuals exposed to such events will develop PTSD [18]. Although previous studies focused on military veterans, there is increasing interest in other groups exposed to accidents such as industrial and motor vehicle accidents. Firefighters represent a population at high risk for the development of PTSD symptoms. In Korea, studies using different diagnostic tools reported PTSD symptoms in 10.4 to 50.9 % of firefighters [4, 19, 20]. The IES-R-K has shown good reliability and validity for assessment of PTSD symptom severity in Korea [17, 21]. In the population studied here, prevalence of PTSD in high-risk group was 10.8 % (with 24/25 cutoff value not shown), however because the study aimed to assess MMPI as related factor of PTSD symptoms for use in programs targeting PTSD prevention, we chose a 17/18 cutoff value for high risk PTSD symptom compared to normal. Because subjects with partial PTSD were included in the positive group in this study, the prevalence of PTSD group in this study of 56.8 % is higher than that in previous studies.

PTSD develops or progresses secondary to chronic exposure to risk factors. Age, sex, social support, alcohol abuse, nature of the traumatic events, psychiatric history, family history of mental disorders, childhood abuse and personality were identified as predictors of PTSD symptoms in previous studies [22–25]. Veterans with a diagnosis of PTSD present with higher rates of several medical conditions (e.g. cancer, stroke, non-fatal heart disease, arthritis) and smoking, and lower frequency of exercise and recommended medical screenings as compared to the age-matched general population [26]. Age and work duration were higher and highly correlated to each other in the positive PTSD subgroup (r = 0.914, p < 0.001). Prevalence of PTSD symptoms did not differ among task subgroups. Older firefighters had been more exposed to job-related traumatic events than younger ones, and job rotation during life-time job history in Korea renders, long-term active firefighters more diversely experienced in job-related events [20]. This is also reflected in previous studies which showed a significant linear relationship between years of experience and levels of traumatic stress in firefighters [27] and that tendency to catastrophize prior to trauma exposure is a significant risk factor for developing posttraumatic stress [28].

We analyzed job stress as an additional factor affecting PTSD. Job stress is a determinant of psychosocial stress among Korean firefighters and correlates with MMPI clinical scales [14, 29]. Occupational stress result in aggressive, hostile and vulnerable personalities and has a negative impact on health behavior [30]. Table 2 shows that high degree KOSS subgroups had higher proportion of PTSD according to job demand, job insecurity, organizational injustice, occupational climate and total score in 2011. Therefore we further analyzed whether MMPI related with PTSD symptoms in stratified subgroups defined according to total KOSS score.

We examined whether personality traits related with the presence of PTSD symptoms following repeated exposure to mentally traumatic events, while controlling for demographic variables and job stress in firefighter during 5 years evaluation. As shown in Table 3, masculinity-femininity scale related PTSD symptoms in overall and lower KOSS subgroups, and social introversion scale related PTSD symptoms in overall and higher KOSS subgroups. Work duration was a statistically significant multivariate factor in the overall population studied. Masculinity-femininity scale and work duration significantly related PTSD symptoms in overall and lower KOSS subgroup while social introversion was factor in higher KOSS subgroup (Table 3). In addition, we analyzed same factors based on KOSS subdomain. Job duration was relating factor with PTSD symptoms in higher job demand (OR 1.062, 95 % CI 1.000–1.128), insufficient job control (OR 1.103, 95 % CI 1.030–1.181), interpersonal conflict (OR 1.060, 95 % CI 1.008–1.114), organizational injustice (OR 1.124, 95 % CI 1.037–1.218), lack of reward (OR 1.065, 95 % CI 1.013–1.120) and lower job insecurity (OR 1.504, 95 % CI 1.050–2.155) as subdomain of KOSS. Social introversion was significant relating factor with PTSD symptom in higher job demand KOSS subdomain (OR 2.766, 95 % CI 1.099–6.964). In case of masculinity-femininity scales, the result was same in higher organizational injustice subdomain (OR 2.752, 95 % CI 1.033–7.326), while hysteria was related in higher job demand (OR 3.042, 95 % CI 1.093–8.466) and insufficient job control subdomain (OR 3.441, 95 % CI 1.110–10.663). These results are partially consistent with the previous finding that higher scores on the MMPI paranoia, hypochondriasis, psychopathic deviate, and masculinity-femininity scales related with PTSD symptoms among male college graduates who later served in the Vietnam War [31]. People with lower social introversion scores are uncomfortable in social interactions, and they simply prefer to be alone. Negative social interactions were found to relate PTSD symptoms after controlling for previous trauma experiences and social support [32]. Bramsen et al. reported that higher scores on a personality measure of negativism predicted subsequent PTSD symptoms among Dutch veterans who participated in the United Nations Protection Force mission in the former Yugoslavia [8]. Negative view of self was associated most strongly with PTSD severity especially among men [33]. Subjects in this study had worked for several years at starting assessment point, therefore social network in work environment likely had been formed. In terms of this specific situation, social isolation was a strong factor of development of PTSD symptoms especially in the high-job stress subgroup of firefighters. This result was consistent with several previous studies. PTSD is maintained by negative appraisals: extreme thoughts of one’s incompetence [10]. Heinrichs reported that recovery form PTSD is significantly influenced by the ability to preserve social support networks and in turn social support might be an important factor for maintaining high levels of self-efficacy in the high-risk population of firefighters [3].

This study has several limitations. First, this study designed case–control with retrospective measures of relating factor of PTSD in experienced firefighters. All participants had already been exposed to several traumatic events before study onset because firefighter with PTSD might be included at starting point and trauma may affect changes in an individual’s personality rendering it difficult to determine whether personality in this sample was an antecedent or a product of PTSD, or whether a bidirectional relation exists between these constructs. And some firefighters suffering PTSD could not be remained in 2011 as healthy worker effect. Second, only one scale, IES-R-K, from a general mental health test was used to identify PTSD. However, IES-R-K has been used extensively in trauma research and for PTSD screening during firefighter annual health examinations in Korea thereby allowing comparisons with other studies. Third, all firefighters in this study were male, limiting ability to generalize results to all firefighters. Fourth, we were unable to consider all factors affecting PTSD symptoms, including number and severity of trauma event and diseases such as depression. The relatively small number of firefighters who completed all questionnaires at both assessment time points might have resulted in selection bias and wide confidence intervals. Future studies including other factors influencing PTSD symptom development and maintenance such as depression and social psychiatric stress, and all firefighters including females are needed to better understand relating factors of PTSD among firefighters.

## Conclusions

Previous studies showed that pre-employment personality traits predicted PTSD symptom development among firefighters and military groups; however, these finding might have limited usefulness for mental health programs for experienced firefighters because of their differing job duration and job stress levels. This study showed that masculinity-femininity and social introversion as well as job duration were significantly associated with PTSD symptoms according to job stress level. Our data suggest that MMPI is a useful screening tool for experienced firefighters and for mental health programs tailored to worksite-environmental related job stress. Further research is needed on the moderating effect of mental health programs based on MMPI for firefighters with repeated exposure to mental hazard.
